# Longitudinal Changes in Serum Interleukin-33 Levels During Acute Psychotic Relapse and Clinical Improvement in Schizophrenia: An Admission-Discharge Study

**DOI:** 10.3390/jcm15197542

**Published:** 2026-09-28

**Authors:** Armando L. Morera-Fumero, José J. Tascón-Cervera, Estefania Diaz-Mesa, Silvia Yelmo-Cruz, Pedro Abreu-González, María Lourdes Fernandez-Lopez

**Affiliations:** 1 Department of Internal Medicine, Dermatology, and Psychiatry, University of La Laguna, 38200 Tenerife, Canary Islands, Spain; jtascer@gobiernodecanarias.org (J.J.T.-C.); ediazmes@ull.edu.es (E.D.-M.); louferna@ull.edu.es (M.L.F.-L.); 2 Department of Psychiatry, Dr. Negrín University Hospital, 35010 Gran Canaria, Canary Islands, Spain; 3 Department of Psychiatry, Canary Islands University Hospital, 38320 Tenerife, Canary Islands, Spain; syelcru@gobiernodecanarias.org; 4 Unit of Physiology, Department of Basic Medical Sciences, University of La Laguna, 38200 Tenerife, Canary Islands, Spain; pabreu@ull.edu.es

**Keywords:** schizophrenia, interleukin-33, IL-33, alarmin, neuroinflammation, psychoneuroimmunology, biomarker, psychotic relapse

## Abstract

**Background/Objectives**: Schizophrenia pathophysiology has been linked to immune dysregulation, but the role of interleukin-33 (IL-33) during acute psychotic relapse remains insufficiently investigated. This exploratory admission–discharge study aimed to evaluate serum IL-33 levels in inpatients with paranoid schizophrenia and in healthy controls. **Methods**: Serum IL-33 was measured at midday (12:00 h) and midnight (24:00 h) in 21 inpatients with paranoid schizophrenia and 21 age- and sex-matched healthy controls (HC). Patients were sampled at admission and discharge. HC underwent one sampling day, with blood collected at both 12:00 h and 24:00 h, during the interval between the patients’ admission and discharge assessments. Psychopathology was assessed using the Positive and Negative Syndrome Scale (PANSS). **Results**: PANSS positive and general psychopathology scores decreased from admission to discharge (*p* < 0.001 and *p* = 0.008, respectively). Serum IL-33 concentrations were descriptively higher in patients than in HC, but between-group differences were not statistically significant. Midnight serum IL-33 increased from admission to discharge (214.9 ± 358.0 vs. 241.2 ± 379.4 pg/mL; *p* = 0.028), whereas the midday change was nonsignificant. Age was inversely correlated with IL-33 in patients; associations with antipsychotic dosage and sex were not statistically significant. **Conclusions**: The midnight increase in IL-33 accompanied clinical improvement, but medication, hospitalization, age, and smoking remain potential confounders. These exploratory findings warrant replication and do not establish IL-33 as a diagnostic or state biomarker.

## 1. Introduction

Schizophrenia (SCZ) is characterized by disturbances in the perception of reality, thought, emotion, behavior, and social and occupational functioning [1]. Its course commonly includes periods of remission and relapse. Functional outcomes vary, although substantial social and functional impairment is common [2].

The neurobiological basis of SCZ remains incompletely understood, but evidence implicates immune dysregulation in its pathophysiology [3]. Altered concentrations of peripheral cytokines, acute-phase proteins, and other inflammatory proteins have been reported during both acute psychotic episodes and clinical stability [4,5,6]. Elevated concentrations of proinflammatory cytokines, including interleukin-2 [7], interleukin-6, interleukin-18 [8], tumor necrosis factor-alpha (TNF-α), and interleukin-1β (IL-1β) [9], have been observed in some patients, suggesting possible relationships with symptom severity, clinical course, or treatment response.

Interleukin-33 (IL-33), a member of the IL-1 cytokine family, is involved in neuroinflammation and neuroimmune signaling [10]. It has cytokine and nuclear functions and can act as an alarmin following cellular damage or stress [10]. IL-33 is expressed in barrier tissues and the central nervous system, including astrocytes, oligodendrocytes, and endothelial cells. Through binding to the membrane-bound ST2 receptor, it influences multiple immune cell populations, including T-helper 2 cells, regulatory T cells, and group 2 innate lymphoid cells [11,12].

Despite evidence linking an IL-33 polymorphism to SCZ risk [13], the potential of IL-33 as a biomarker remains insufficiently investigated, particularly during acute psychotic relapse. Findings are inconsistent: some studies have reported higher circulating IL-33 concentrations in patients than in healthy participants [14,15], whereas others have found no significant between-group differences [14,16,17,18].

A recent systematic review of IL-33 in SCZ [19] described a small and heterogeneous evidence base, with substantial variation in clinical status, study design, and psychopathological assessment. It also identified a lack of longitudinal studies specifically examining acute psychotic relapse.

Examining IL-33 concentrations during acute relapse and subsequent clinical improvement may help clarify their relationship with clinical state. This study investigated serum IL-33 concentrations at midday and midnight in patients hospitalized for an acute psychotic relapse, comparing admission with discharge and patients with HC.

## 2. Methods

### 2.1. Participants

Patients were initially selected in the emergency department of the Canary Islands University Hospital because they presented with acute psychotic decompensation requiring admission to the psychiatric ward. Selection in the emergency department was not based on a diagnosis of paranoid schizophrenia. After admission, two experienced psychiatrists evaluated patients using the Structured Clinical Interview for the Diagnostic and Statistical Manual of Mental Disorders, Fourth Edition, Text Revision (DSM-IV-TR [1]). Five patients were excluded after this evaluation because they did not meet diagnostic criteria for schizophrenia. The final cohort comprised 21 inpatients diagnosed with the DSM-IV-TR paranoid subtype. The International Classification of Diseases, Tenth Revision (ICD-10) code F20.0 is provided for diagnostic correspondence; the WHO clinical descriptions and diagnostic guidelines were published in 1992 [20].

Patients were recruited consecutively over six months (January–June 2009). All eligible patients admitted during this period who consented to participate were included. No a priori sample-size calculation was performed. The study is therefore exploratory, and its limited sample size restricts statistical power and generalizability.

Twenty-one HC, matched to patients by age and sex and with no personal or family psychiatric history, were recruited from acquaintances of the researchers. Physical health was assessed through a brief medical history and routine laboratory testing. Psychiatric history was assessed by asking about past or current psychiatric or psychological treatment in participants and their first-degree relatives.

Eligible patients were aged 18–65 years and able to speak and understand Spanish. Exclusion criteria were intellectual disability; a history of severe trauma or head injury; alcohol or substance abuse; a physical medical condition; pregnancy; use of anti-inflammatory, immunosuppressive, or antiviral medication; physical agitation; current infection; and autoimmune or metabolic disorders.

### 2.2. Diagnostic Work-Up and Assessment of Potential Secondary Causes

Diagnostic assessment included psychiatric and medical histories, clinical examination, assessment of physical health, and routine blood tests obtained as part of clinical care. All included patients met the prespecified eligibility criteria.

Magnetic resonance imaging (MRI), electroencephalography (EEG), lumbar puncture, formal neuropsychological assessment, extended etiological testing, and urine toxicology were not mandated by the study protocol or systematically recorded for every participant. These investigations were requested when clinically indicated by the history, examination, or suspected medical or neurological conditions.

The available data therefore do not support retrospective classification into primary schizophrenia and secondary psychotic-syndrome groups on the basis of MRI, EEG, or lumbar-puncture findings.

### 2.3. Clinical Assessment

Psychopathology was assessed using the Spanish adaptation of the Positive And Negative Syndrome Scale (PANSS) [21,22]. The scale comprises 30 clinician-rated items covering positive symptoms (7 items), negative symptoms (7 items), and general psychopathology (16 items), each rated from 1 (absent) to 7 (extreme). These domains describe the scale’s clinical content; content validity was not independently assessed in this study. In a validation study of 100 Spanish patients with schizophrenia, Peralta Martín and Cuesta Zorita reported good interrater reliability, appropriate construct validity, and high criterion-related validity against the SAPS and SANS, supporting convergence with established symptom measures. They also reported modest internal consistency and insufficient factorial validity for the positive subscale [21]. These findings come from the published validation study and are not psychometric estimates from the present cohort.

### 2.4. Study Protocol

Blood samples were collected between January and June 2009. Patients were assessed on two sampling days: the day after admission and the day before discharge. HC were assessed on a single day between the patients’ admission and discharge assessments. Blood was collected at 12:00 h and 24:00 h on each sampling day, yielding four scheduled collections per patient and two per HC. These times were selected to minimize disruption to hospital routines; two measurements 12 h apart cannot characterize a complete circadian rhythm. Participants rested in bed for one hour before each collection to reduce sampling-related stress. Blood was collected into Vacutainer tubes without anticoagulant, allowed to clot, and centrifuged at 3000 rpm for 5 min. Serum was separated, aliquoted into Eppendorf tubes, and stored at −70 °C. Samples were thawed only once, in summer 2021, for IL-33 analysis.

The study was conducted in accordance with the Declaration of Helsinki. All participants provided written informed consent before inclusion. The Ethics and Investigation Committee of the Canary Islands University Hospital approved the protocol (No. 2008-41).

### 2.5. Measurement of Interleukin-33

Serum IL-33 concentrations were measured using a solid-phase ELISA (Millipore, Saint Louis, MO, USA) according to the manufacturer’s instructions. The calibration range was 0–500 pg/mL. Analytical controls and samples with IL-33 concentrations above the upper limit of quantification (>500 pg/mL) were diluted 1:2 before analysis. The dilution procedure had been validated for linearity and recovery within the 0–500 pg/mL range. Absorbance was measured at 450 nm using a SpectraMax 190 microplate spectrophotometer (Molecular Devices, Sunnyvale, CA, USA). A one-phase exponential-decay model provided the best fit to the standard curves (R^2^ = 0.9967–0.9990). The limit of detection was 1.202 pg/mL. The intra-assay and inter-assay coefficients of variation were 4.09% and 10.03%, respectively.

To minimize analytical variability, all samples were analyzed on the same day in summer 2021 by the same analyst. The analyst was blinded to group, clinical assessment (admission or discharge), and sampling time (midday or midnight).

### 2.6. Statistical Analysis

Data were analyzed using IBM SPSS Statistics for Windows, version 29.0 (IBM Corp., Armonk, NY, USA). Smoking-related analyses were independently verified using Python version 3.12.14 and SciPy version 1.17.0.

Between-group differences in serum IL-33 concentrations were examined using independent-samples *t*-tests, with Welch’s unequal-variance correction used for patient–HC comparisons. Within-patient differences between admission and discharge and between 12:00 h and 24:00 h were examined using paired-samples *t*-tests. Associations between categorical variables were examined using chi-square tests. Relationships between continuous variables were assessed using Pearson correlations, with age-adjusted partial correlations where specified. Continuous variables are presented as mean ± standard deviation (SD), and categorical variables as counts or percentages. All tests were two-tailed, with statistical significance set at *p* < 0.05. Age was retained as a continuous variable; no age-stratified analyses were performed. Age adjustment was restricted to clinical correlations within patients; patient–HC contrasts were unadjusted. Smoking-status comparisons excluded participants with missing smoking data (two patients and one HC), without imputation. The between-group comparison used Yates’ continuity-corrected chi-square, with a two-tailed Fisher exact test as a sensitivity analysis. Within patients, IL-33 concentrations were compared between smokers and nonsmokers using two-tailed independent-samples Student *t*-tests with pooled variance; Welch tests were used as a sensitivity analysis. These exploratory comparisons included 19 patients with known smoking status, and their *p* values were unadjusted for multiple comparisons.

Antipsychotic doses were converted to chlorpromazine equivalents (CPZE) to facilitate comparisons across treatments [23,24].

## 3. Results

### 3.1. Participant Description and Assessment of Potential Secondary Causes

Five patients initially selected during acute psychotic decompensation were excluded after inpatient diagnostic evaluation because they did not meet DSM-IV-TR criteria for schizophrenia. The final sample included 21 HC and 21 inpatients diagnosed with the DSM-IV-TR paranoid subtype who met the study eligibility criteria. Routine clinical assessment and laboratory testing were used to identify relevant medical conditions. As described in Section 2.2, extended neurological and etiological investigations were not systematic, precluding retrospective classification into primary and secondary psychotic-syndrome groups.

Sociodemographic and clinical characteristics are presented in Table 1. Smoking status was available for 19 patients and 20 HC. Seven patients were smokers and 12 were nonsmokers; three HC were smokers and 17 were nonsmokers. Smoking status was missing for two patients and one HC. Among participants with known status, smoking prevalence was 36.8% in patients and 15.0% in HC. The between-group difference was not statistically significant (Yates-corrected chi-square = 1.427, *p* = 0.232; two-tailed Fisher exact *p* = 0.155), but this does not establish comparable tobacco exposure or exclude confounding. Age and sex differences were also not statistically significant.

Table 2 presents PANSS subscale scores at admission and discharge. Positive and general psychopathology scores decreased from admission to discharge (*p* < 0.001 and *p* = 0.008, respectively). The change in negative scores was not statistically significant (*p* = 0.125).

### 3.2. Serum IL-33 Concentrations and Participant Characteristics

#### 3.2.1. Healthy Controls

In HC, we examined the relationships of serum IL-33 concentrations with age, sex, BMI and sampling time (12:00 h and 24:00 h). Age was inversely correlated with serum IL-33 in HC at 12:00 h but the correlation did not reach statistical significance (r = −0.089, *p* = 0.718) and 24:00 h (r = −0.208, *p* = 0.365). No significant sex differences were observed at 12:00 h (men: 106.1 ± 164.1 pg/mL; women: 299.9 ± 389.7 pg/mL; *p* = 0.334) or 24:00 h (men: 92.3 ± 179.7 pg/mL; women: 287.3 ± 381.9 pg/mL; *p* = 0.324). Concentrations did not differ significantly between 12:00 h (152.2 ± 240.3 pg/mL) and 24:00 h (138.7 ± 246.3 pg/mL; *p* = 0.49). BMI was inversely correlated with serum IL-33 in HC at 12:00 h but the correlation did not reach statistical significance (r = −0.137, *p* = 0.689) and 24:00 h (r = −0.267, *p* = 0.428). Three HC were smokers, 17 were nonsmokers, and one had missing smoking status. Smoking-stratified IL-33 comparisons were not performed in HC because only three were smokers.

#### 3.2.2. Patients

Serum IL-33 concentrations did not differ significantly by sex at either assessment or sampling time (Table 3).

Table 4 presents the correlation between age and IL-33 as well as with BMI. Contrary to the HC, older age was associated with lower serum IL-33 concentrations in patients at both admission and discharge and at both sampling times. BMI was not associated with IL-33 in patients at both admission and discharge and at both sampling times.

Serum IL-33 concentrations did not differ significantly between 12:00 h and 24:00 h at admission or discharge (Table 5).

Smoking-stratified analyses included 19 patients with known smoking status (7 smokers and 12 nonsmokers). The two patients with missing smoking data were excluded only from these comparisons and remained in the overall IL-33 summaries. Serum IL-33 concentrations did not differ significantly by smoking status at admission or discharge at either sampling time (Table 6). Welch sensitivity tests were also nonsignificant (*p* = 0.475, 0.453, 0.465, and 0.451 for admission at 12:00 h and 24:00 h and discharge at 12:00 h and 24:00 h, respectively). These small, unadjusted comparisons cannot exclude a smoking effect.

Table 7 presents age-adjusted partial correlations between serum IL-33 concentrations and clinical variables in patients with SCZ. None of the reported correlations was statistically significant. Age adjustment applies only to clinical correlations in the patient group.

### 3.3. Comparisons Between Patients and Healthy Controls

**Figure 1:**
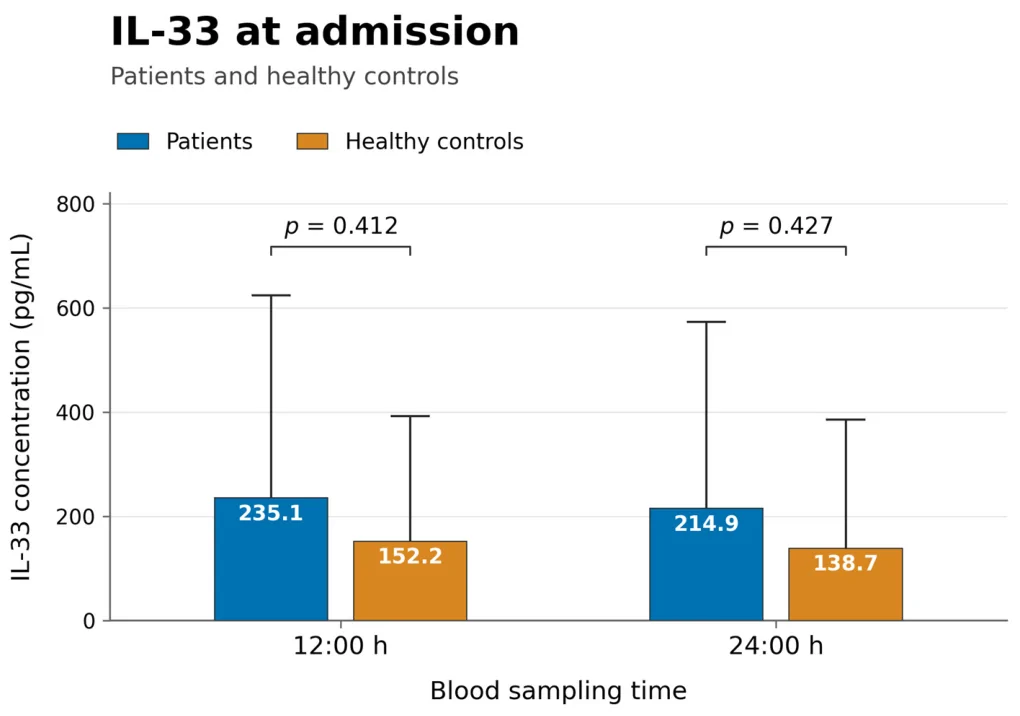
Serum IL-33 concentrations (pg/mL) in patients at admission and healthy controls at 12:00 h and 24:00 h. Bars represent means; upper error bars represent one SD above the mean. Each group includes 21 participants. Brackets show unadjusted two-tailed independent-samples Welch’s *t*-test *p* values. Healthy controls were assessed on one sampling day and provide the same cross-sectional reference in Figure 1 and Figure 2. IL-33, interleukin-33; SD, standard deviation.

**Figure 2:**
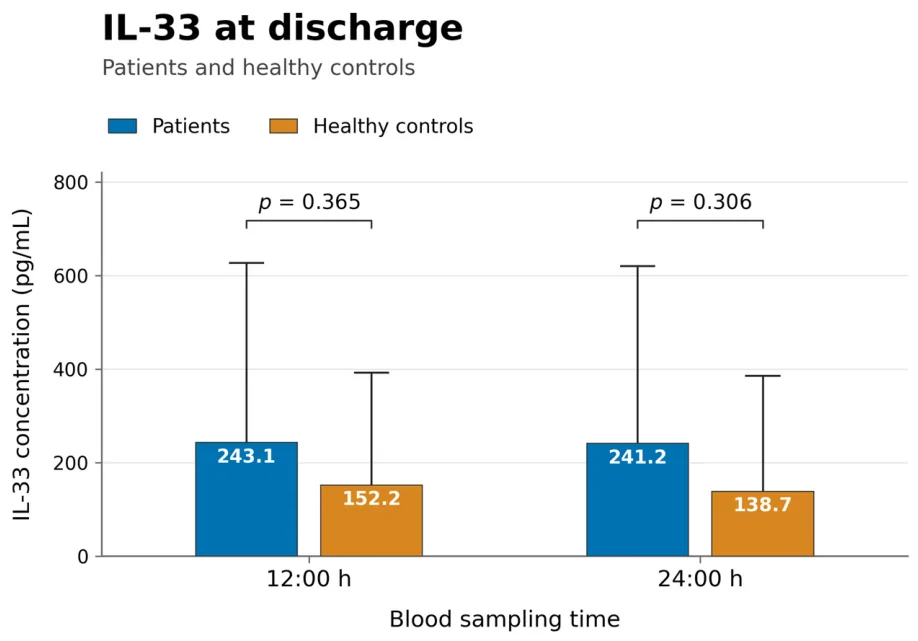
Serum IL-33 concentrations (pg/mL) in patients at discharge and healthy controls at 12:00 h and 24:00 h. Bars represent means; upper error bars represent one SD above the mean. Each group includes 21 participants. Brackets show unadjusted two-tailed independent-samples Welch’s *t*-test *p* values. The healthy-control summaries are the same as in Figure 1; controls were not followed longitudinally. IL-33, interleukin-33; SD, standard deviation.

Mean serum IL-33 concentrations were higher in patients than in HC at both assessments and sampling times (Figure 1 and Figure 2). None of the four comparisons was statistically significant using two-tailed independent-samples Welch’s *t*-tests: admission at 12:00 h, *p* = 0.412; admission at 24:00 h, *p* = 0.427; discharge at 12:00 h, *p* = 0.365; and discharge at 24:00 h, *p* = 0.306. These *p* values are unadjusted for multiple comparisons.

### 3.4. Changes from Admission to Discharge

Serum IL-33 concentrations increased from admission to discharge at 24:00 h (*p* = 0.028). The increase at 12:00 h was not statistically significant (*p* = 0.360; Table 8).

## 4. Discussion

This exploratory longitudinal study examined serum IL-33 during acute psychotic relapse and subsequent clinical improvement in SCZ. Mean concentrations were higher in patients than in HC, although none of the between-group comparisons was statistically significant. Within patients, IL-33 increased from admission to discharge at midnight, whereas the midday change was not significant. These findings do not establish a persistent between-group difference in IL-33 or validate IL-33 as a state biomarker.

Previous studies have reported both higher IL-33 concentrations in patients than in controls [14,15] and no significant between-group differences [16,17,18]. A longitudinal study [25] assessed IL-33 after three months of antipsychotic treatment without a healthy control comparison and found no statistically significant change. In the present study, age-adjusted correlations between CPZE and serum IL-33 were not significant. Other studies have also reported no significant association between antipsychotic treatment and IL-33 [16,17]. These findings do not rule out medication effects.

Age was inversely correlated with serum IL-33 concentrations in both groups. Although age-related immune changes have been described in schizophrenia [26], these cross-sectional correlations do not establish an aging mechanism. Age was retained as a continuous variable, and clinical correlations were adjusted for age. Dividing this small sample into age categories would reduce precision and make the results dependent on the chosen cutoff. Nonlinear age effects and age-related confounding of the unadjusted patient–HC comparisons remain possible. Larger studies are needed to examine these issues. Similarly, nonsignificant sex and sampling-time comparisons do not establish the absence of such effects.

The increase in serum IL-33 at midnight occurred alongside improvements in PANSS positive and general psychopathology scores. However, the reported correlations do not assess whether changes in IL-33 tracked within-person changes in symptoms, and no significant correlations with the clinical variables examined were found. IL-33 may have proinflammatory or neuroprotective effects depending on its biological context and target cells [27,28]. The present findings support further longitudinal investigation but do not demonstrate normalization toward HC concentrations. The nonsignificant decrease reported after three months of outpatient treatment by Subbanna et al. [25] illustrates the heterogeneity of the available evidence.

These findings should be considered within the broader evidence of altered cytokine networks in SCZ. Meta-analyses have reported changes in peripheral proinflammatory cytokines, including interleukin-6, TNF-α, and IL-1-related molecules, particularly during acute psychotic states and in antipsychotic-naïve patients [4]. IL-33 may participate in these networks through effects on glial activation, microglia–neuron communication, and synaptic function [27]. Nevertheless, the nonsignificant patient–HC comparisons in this study do not establish a reliable between-group difference in serum IL-33.

The hypothesis that lower serum IL-33 could reflect less tissue injury is not directly tested by these data. Mean IL-33 concentrations were descriptively higher in patients than in HC, and the significant longitudinal change was an increase at midnight. Neither a lower nor a higher serum concentration establishes the extent of tissue injury: concentrations may reflect differences in release, production, clearance, or receptor binding, and peripheral measurements do not establish the direction of effects within the central nervous system. In mice, Nguyen et al. showed that neuronal IL-33 promotes microglial engulfment of extracellular matrix and supports synaptic plasticity [29]. These findings provide a rationale for considering altered homeostatic signaling alongside injury-related explanations, but they do not demonstrate either mechanism in schizophrenia. The present study cannot determine whether the serum pattern reflects tissue injury, compensatory signaling, or other processes, and it provides no direct evidence of reduced injury or neuroprotection.

Several limitations require consideration. The small sample limits statistical power and precision, and nonsignificant between-group differences do not demonstrate equivalence. Follow-up ended at discharge, preventing assessment of longer-term trajectories or relapse prediction. Serum IL-33 may not reflect central nervous system signaling, and sST2 was not measured. IL33 mRNA expression was not assessed, so altered transcription cannot be distinguished from other determinants of serum protein concentrations. Future studies should combine cellular transcript measurements with IL-33 protein and sST2 measurements. Extended etiological investigations were not systematic, precluding retrospective stratification by potential secondary causes.

Patients were recruited in 2009 through an emergency department during acute psychotic decompensation requiring hospitalization. The paranoid subtype describes the final cohort after inpatient diagnostic evaluation; it was not a criterion for initial selection in the emergency department. Hospital-based epidemiology provides relevant context. In a large ICD-10-based inpatient dataset from China, paranoid schizophrenia was the most frequent specifically identified subtype, although many records had no specified subtype [30]. This describes the diagnostic composition of an inpatient sample rather than the relative risk of admission across subtypes. Positive symptoms predicted hospitalization in a first-episode psychosis cohort [31], while a national epidemiological study also associated negative symptoms with hospitalization [32]. These studies support the relevance of examining acute psychotic presentations but do not establish a universal ranking of hospital need by subtype.

DSM-5 and ICD-11 replaced the classical schizophrenia subtypes with dimensional descriptions of psychopathology [33,34]. Clinically, the present cohort represents schizophrenia during acute psychotic exacerbation requiring inpatient care, but patients were not reassessed using these newer diagnostic criteria. The emergency and inpatient recruitment setting and the diagnostic composition of the final cohort limit generalizability. Consecutive recruitment does not eliminate selection bias, and the findings should not be generalized to predominantly negative-symptom presentations or other schizophrenia populations. PANSS negative scores within this cohort cannot substitute for a comparison with a separately recruited group with predominantly negative symptoms. Whether such a group would have different IL-33 concentrations, and in which direction, remains unknown.

Prolonged storage is an additional preanalytical limitation. Samples were stored at −70 °C for approximately 12 years and thawed once for analysis. Published evidence indicates that storage effects depend on the analyte and storage conditions. Doumatey et al. found no significant change in high-sensitivity C-reactive protein (hs-CRP) concentrations in 30 serum samples after 7–11 years at −80 °C [35]. In the Janus Serum Bank, comparisons of serum groups stored for one month, two years, and 25 years at −25 °C showed small or nonsignificant differences in albumin, IgG, and IgE concentrations [36]. These findings concern measured concentrations and do not establish preservation of molecular structure.

Evidence concerning other proteins does not establish IL-33 stability during approximately 12 years of storage at −70 °C. Stability under these conditions was not evaluated in this study, and the effect of prolonged storage on measured IL-33 concentrations remains uncertain.

HC were sampled on a single day and were not followed under the same inpatient conditions as patients. Resting for one hour before blood collection standardized the immediate sampling conditions but could not control for the effects of hospitalization, sleep, activity, diet, or changes in stress during admission. No comparison group was followed under equivalent inpatient conditions. Antipsychotic treatment was not randomized, and no antipsychotic-exposed healthy control group was included. Nonsignificant correlations with CPZE do not exclude medication effects or separate them from clinical improvement and the inpatient environment. Among participants with recorded smoking status, prevalence was 36.8% in patients and 15.0% in HC; smoking data were missing for two patients and one HC. The nonsignificant smoking-status comparison does not establish comparable exposure. Smoking-stratified IL-33 comparisons involved only 19 patients and were nonsignificant at all four assessments. The small subgroup sizes, missing smoking data, and lack of adjustment for potential confounders limit interpretation; these results do not exclude a smoking effect or residual confounding.

Multiple exploratory comparisons increase the risk of chance findings. A significant change at midnight and a nonsignificant change at midday do not, by themselves, establish an interaction between sampling time and assessment. The longitudinal finding requires independent replication and joint analysis of both sampling times in a sufficiently large cohort.

Strengths include repeated sampling within participants from admission to discharge, standardized midday and midnight collections, and blinded analysis of all samples in a single assay batch. The study also examined age and antipsychotic dose and addressed a cytokine that remains insufficiently studied in schizophrenia. These features support further investigation of the longitudinal pattern, while the small sample and nonsignificant patient–HC comparisons require cautious interpretation. The heterogeneous literature on IL-33, sST2, and the IL-33/sST2 axis reinforces the need for replication [19].

Serum IL-33 concentrations were descriptively higher in patients than in HC, but the between-group differences were not statistically significant. IL-33 increased from admission to discharge at midnight, whereas the midday change was not significant. This exploratory longitudinal finding warrants replication but is insufficient to establish IL-33 as a diagnostic or state biomarker. Future studies should include larger samples, longer follow-up, age adjustment, standardized assessment of secondary causes, and concurrent sST2 measurement.

## Figures and Tables

**Table 1:** Sociodemographic and clinical characteristics of patients and healthy controls.

Characteristic	Patients	Healthy Controls	*p*
Age, years	39.2 ± 13.3	36.6 ± 12.1	0.520
Sex, men/women	15/6	16/5	0.730
Smoking, yes/no/missing	7/12/2	3/17/1	0.232
CPZE, mg/day	778.4 ± 485.0	—	—
Age at illness onset, years	19.7 ± 4.9	—	—
Illness duration, years	18.8 ± 13.9	—	—
Previous hospitalizations, n	5.8 ± 4.7	—	—
Length of hospitalization, days	20.7 ± 10.1	—	—

Values are mean ± SD or counts. Patient and HC groups each comprise 21 participants. Smoking-status comparisons include 19 patients and 20 HC with known status; two patients and one HC had missing smoking data. Smoking percentages use the number with known status as the denominator. The tabulated smoking *p* value is from Yates’ continuity-corrected chi-square; two-tailed Fisher exact *p* = 0.155. CPZE, chlorpromazine equivalent dose (mg/day); HC, healthy controls; SD, standard deviation. A dash indicates that a variable or comparison is not applicable.

**Table 2:** PANSS subscale scores at admission and discharge.

PANSS Subscale	Admission	Discharge	*p*
Positive	19.00 ± 5.96	11.62 ± 4.12	<0.001
Negative	14.23 ± 5.67	11.38 ± 3.71	0.125
General psychopathology	23.87 ± 4.34	19.93 ± 3.45	0.008

Values are mean ± SD. PANSS, Positive And Negative Syndrome Scale; SD, standard deviation. *p* values are from two-tailed paired-samples *t*-tests using complete pairs.

**Table 3:** Serum IL-33 concentrations in patients by sex, assessment, and sampling time.

Assessment and Time	Men	Women	*p*
Admission, 12:00 h	213.6 ± 394.1	288.7 ± 407.3	0.700
Admission, 24:00 h	200.1 ± 380.8	249.8 ± 323.4	0.780
Discharge, 12:00 h	220.9 ± 393.1	298.5 ± 389.9	0.690
Discharge, 24:00 h	215.2 ± 386.1	306.2 ± 388.9	0.630

Values are mean ± SD in pg/mL. IL-33, interleukin-33; SD, standard deviation. *p* values are from two-tailed independent-samples *t*-tests comparing men and women.

**Table 4:** Correlations between age and Body Mass Index (BMI) and serum IL-33 concentrations in patients.

Assessment	Sampling Time	r	*p*
Age, Admission	12:00 h	−0.476	0.040
Age, Admission	24:00 h	−0.483	0.036
Age, Discharge	12:00 h	−0.487	0.034
Age, Discharge	24:00 h	−0.514	0.024
BMI, Admission	12:00 h	0.113	0.757
BMI, Admission	24:00 h	0.102	0.780
BMI, Discharge	12:00 h	0.181	0.618
BMI, Discharge	24:00 h	0.213	0.554

IL-33, interleukin-33; r, Pearson correlation coefficient. *p* values are two-tailed.

**Table 5:** Midday and midnight serum IL-33 concentrations in patients.

Assessment	12:00 h	24:00 h	*p*
Admission	235.1 ± 389.1	214.9 ± 358.0	0.087
Discharge	243.1 ± 384.1	241.2 ± 379.4	0.750

Values are mean ± SD in pg/mL. IL-33, interleukin-33; SD, standard deviation. *p* values are from two-tailed paired-samples *t*-tests comparing 12:00 h with 24:00 h within each assessment.

**Table 6:** Serum IL-33 concentrations by smoking status in patients with schizophrenia.

Assessment and Time	Nonsmokers (n = 12)	Smokers (n = 7)	*p*
Admission, 12:00 h	304.2 ± 434.7	167.9 ± 363.0	0.495
Admission, 24:00 h	279.4 ± 403.5	148.7 ± 326.0	0.477
Discharge, 12:00 h	312.8 ± 424.0	173.7 ± 368.1	0.480
Discharge, 24:00 h	311.0 ± 426.1	172.0 ± 346.7	0.475

Values are mean ± SD in pg/mL. The same 19 patients contribute to all four comparisons. Two patients had missing smoking status and were excluded from these subgroup analyses without imputation. *p* values are unadjusted, two-tailed independent-samples Student *t*-tests with pooled variance (17 degrees of freedom). IL-33, interleukin-33; SD, standard deviation.

**Table 7:** Age-adjusted correlations between serum IL-33 concentrations and clinical variables in patients.

Clinical Variable	Admission 12:00 h	Admission 24:00 h	Discharge 12:00 h	Discharge 24:00 h
Age at illness onset	0.174 (0.610)	0.216 (0.524)	0.196 (0.563)	0.213 (0.530)
Illness duration	−0.083 (0.807)	−0.119 (0.728)	−0.102 (0.766)	−0.116 (0.734)
Previous hospitalizations	0.510 (0.109)	0.500 (0.117)	0.492 (0.124)	0.499 (0.118)
Length of hospitalization	−0.156 (0.647)	−0.148 (0.665)	−0.148 (0.663)	−0.114 (0.738)
CPZE	0.206 (0.414)	0.216 (0.406)	0.202 (0.417)	0.228 (0.396)
PANSS positive, admission	−0.240 (0.477)	−0.290 (0.388)	−0.264 (0.433)	−0.271 (0.420)
PANSS negative, admission	0.129 (0.705)	0.207 (0.542)	0.146 (0.669)	0.174 (0.609)
PANSS general, admission	−0.211 (0.534)	−0.226 (0.503)	−0.219 (0.517)	−0.216 (0.524)
PANSS positive, discharge	0.155 (0.649)	0.078 (0.820)	0.134 (0.695)	0.105 (0.759)
PANSS negative, discharge	−0.071 (0.836)	−0.080 (0.816)	−0.103 (0.762)	−0.064 (0.852)
PANSS general, discharge	−0.320 (0.337)	−0.322 (0.335)	−0.325 (0.329)	−0.288 (0.390)

Each cell shows the age-adjusted partial correlation coefficient r, with its two-tailed *p* value in parentheses on the second line. Analyses include patients with schizophrenia only. CPZE, chlorpromazine equivalent dose; IL-33, interleukin-33; PANSS, Positive And Negative Syndrome Scale. Admission and discharge in the row labels identify the timing of PANSS assessment.

**Table 8:** Changes in serum IL-33 concentrations from admission to discharge.

Sampling Time	Admission	Discharge	*p*
12:00 h	235.1 ± 389.1	243.1 ± 384.1	0.360
24:00 h	214.9 ± 358.0	241.2 ± 379.4	0.028

Values are mean ± SD in pg/mL. IL-33, interleukin-33; SD, standard deviation. *p* values are from two-tailed paired-samples *t*-tests comparing admission with discharge at each sampling time.

## Data Availability

The original contributions presented in this study are included in the article. Further inquiries can be directed to the corresponding author.
